# Analysis of Electrical Conductivity in Commercial Adhesives Incorporating Graphene Nanoplatelets for Industrial Applications

**DOI:** 10.3390/polym17010047

**Published:** 2024-12-28

**Authors:** Pablo Rodríguez Fernández, Cristina Alía García

**Affiliations:** E.T.S. de Ingeniería y Diseño Industrial, Universidad Politécnica de Madrid (España), 28040 Madrid, Spain; pablo.rfernandez@alumnos.upm.es

**Keywords:** electrical conductivity, epoxy, polyurethane, composite, adhesives, graphene nanoplatelets, GNPs, percolation, sonication, mechanical properties

## Abstract

Polymers are often insulators, but this not a universal intrinsic characteristic of all polymers. For this work, the adhesives used, epoxy and polyurethane, do demonstrate this insulating characteristic. However, there has been significant interest in the development of conductive polymers, specifically adhesives, because of the potential properties and ease of processing of these polymers. The electrical-conductivity values for two composites reinforced with graphene nanoplatelets (GNPs) were measured. Both matrices are intended for industrial usage. One composite used an epoxy matrix, while the other employed a polyurethane resin as the matrix. To achieve dispersion and exfoliation of the filler, the catalyst for each resin, mixed with the GNP in proportion, was subjected to an ultrasonic bath for 30 min. The molds were filled by gravity, with the polyurethane specimens leveled to improve surface finish. The two-point uniaxial method was used to measure the conductivity of the specimens at room temperature, both before and after annealing at 120 °C for 120 min. Conductivity values were obtained for all samples, showing an increase after annealing; however, this increase was less pronounced compared to similar studies. The time and power in the ultrasonic bath, as well as the annealing conditions, must be optimized and the electrical conductivity should be studied periodically.

## 1. Introduction

Polymers are often insulators, but this is not a universal intrinsic characteristic of all polymers. For this work, the adhesives used, epoxy and polyurethane, do demonstrate this insulating characteristic. However, there has been significant interest in the development of conductive polymers, specifically adhesives. This development has the potential to transform the electrical and electronics industries by combining the mechanical and chemical properties, ease of processing, and low cost associated with polymers, with the ability to conduct electricity. In this context, epoxy resins stand out as the adhesives most commonly used as matrices. On the other hand, polyurethane resins share similarities with epoxy resins, but are characterized by greater flexibility and elasticity.

Most adhesives can acquire conductive properties by the addition of conductive fillers, although this approach has certain limitations, primarily economic ones. This approach is largely associated with procedures used to improve the dispersion of nanofillers, especially when the filler percentage is high, and the cost of fillers such as graphene and its derivatives. There are a multitude of studies on adhesives and polymers with conductive fillers, all with varying nanofiller concentrations, curing parameters, dispersion methods, sample processing, etc. Therefore, no consensus seems to have been reached on the optimization of fillers and procedures. In response to this, the use of GNPs is being explored; these share characteristics with graphene, but are simpler to obtain and thus more economical. S. Prolongo and collaborators, in 2022 [1], studied the influence of the surface area of the GNPs in epoxy resin composites, where it was found that a higher specific area results in greater conductivity in the transverse direction of the samples. On the other hand, there were indications that a longer time under ultrasonic bath for filler dispersion and graphene exfoliation leads to a more homogeneous dispersion of GNPs throughout the Section 2, but also greater particle breakage, resulting in lower specific area and worse conductivity. Regarding polyurethane matrix composites, Jorge Canales and his team [2], in 2016, used reduced graphene oxide as a filler instead of GNPs. For the post-curing step at 120 °C, the curing parameters recommended by other studies under similar conditions were considered to ensure the reproducibility and comparability of the results [2].

The ultrasonic bath process was designed with a balanced approach to achieve adequate dispersion of GNPs while avoiding potential fragmentation, which could reduce their surface area and, consequently, the composite’s conductivity. A duration of 30 min was selected based on previous studies suggesting that shorter times may be insufficient for achieving homogeneous dispersion, while excessively long durations could damage the GNPs due to the mechanical energy applied. Although the literature reports varying times ranging from 10 min to over an hour, these times depend on factors such as the type of GNPs, the viscosity of the matrix, and the equipment used. In this study, 30 min was chosen as an intermediate duration, ensuring good initial dispersion without significantly compromising the integrity of the GNPs. In Prolongo’s results [1], it can be observed that the highest conductivity is achieved at a time of 30 min, after which it decreases to a minimum due to filler fragmentation and then increases again, driven by a more homogeneous dispersion.

The objective of this work is to propose the use of two commercially available adhesives commonly used in industry as matrices, an epoxy resin and a polyurethane resin, and GNPs as the filler, focusing on identifying optimal processing conditions to enhance their conductivity and industrial applicability. The effects of the ultrasonic bath time for filler dispersion and exfoliation were explored, aiming to reduce this time while simultaneously improving conductivity, following the conclusions of the scientific literature on this topic. Additionally, the influence of a second curing or annealing step at a high temperature on the electrical conductivity of the samples was analyzed, based on conclusions observed in other studies [2,3]. The additional thermal curing process (120 °C for 120 min) improved conductivity in both matrices, with polyurethane showing more consistent enhancements, likely due to its intrinsic flexibility and compatibility with these conditions. Conversely, epoxy allowed for higher GNP concentrations (up to 40% by volume) but was more susceptible to fabrication defects, such as air bubbles, which reduced its electrical performance. These findings suggest that polyurethane is more suitable for applications requiring stable conductivity and simpler processing, while epoxy may be viable for specific uses where higher filler content is advantageous. It was found that a shorter time probably leads to poorer dispersion at high filler concentrations, as significant variability is shown in the conductivity values, likely due to the increased viscosity of the mixture, which aligns with the results of S. Prolongo [1].

The main contribution of this article lies in the integration of comparative analysis of epoxy and polyurethane matrices in commercial adhesives with GNPs, simultaneously evaluating dispersion, curing, and practical applications. This offers a more complete and practical perspective for the design of conductive adhesives in the industry. In addition, it investigates the effects of filler concentration on commercial adhesives, specifically Sikadur and Sikaforce, evaluating the impacts of varying GNP concentrations (0–40% by volume for epoxy and 0–25% for polyurethane). This broader concentration range enables a more thorough analysis of the practical limitations imposed by increasing GNP content, particularly concerning material viscosity, while exploring the achievable conductivities at low filler percentages, the most economically interesting scenario, which provides a relevant industrial perspective.

Six specimens were manufactured using the polyurethane resin for each of the following GNP volume concentrations: 0, 5, 10, 15, 20, and 25%; six specimens were also created for each selected concentration in the epoxy resin: 0, 5, 10, 15, 20, 25, and 40%. In total, 78 samples were produced.

## 2. Materials and Methods

The epoxy resin used is the SKADUR 52 INJECTION LP (EPOXY) model from the Sikadur brand. It is a low-viscosity, two-component, solvent-free injection resin, designed for filling cracks in concrete, mortar, wood, and similar materials (Table 1).

For the polyurethane resin, the model used is SIKAFORCE-422 L45, a two-component, solvent-free, waterproof urethane adhesive, usually applied to bond sandwich panels and other construction materials (Table 1).

The molds used for the specimens were polystyrene Petri dishes, including both a base and lid.

After demolding, each specimen had its diameter and thickness measured individually.

The resins used do not require high-temperature curing, only room temperature. However, post-curing was conducted at 120 °C for both adhesives for 120 min to assess its effect on electrical conductivity, following the methodology of Jorge Canales and the other relevant literature [2,4].

To demold the specimens, the DEMOULDANT 841 release agent from Sika was used. This is a solvent-based wax for non-porous surfaces, as in this case. Each mold was coated with three layers of solvent using a brush, allowing one minute for drying between layers.

GNPs from the Avanzare brand (Table 2), model av-PLAT-7, were used as filler in the matrix, with the following properties (see Appendix B):

An ultrasonic cleaner from the IBX brand, with a 3.2 L capacity and 120 W of ultrasonic power, was used for exfoliating the nanoplatelets.

Finally, a convection oven was used for the desired thermal treatment of samples after room-temperature curing. The oven was a Selecta model with a power of 3000 W and a temperature adjustable from 40 °C to 300 °C.

The manufacturing process for the specimens proceeded as follows for both resins. First, the mass equivalent of each GNP volume percentage was calculated and weighed on a balance. The appropriate amount of the less viscous resin component was also weighed before mixing with the filler. The mixture was stirred vigorously with rods for one minute until homogeneous and then subjected to an ultrasonic bath for 30 min at 120 W. Bath temperature was not controlled during this test. Once the mixture was dispersed and graphene exfoliated, the corresponding amount of the second resin component was added, and the mixture was stirred with rods for three minutes.

**Figure 1:**
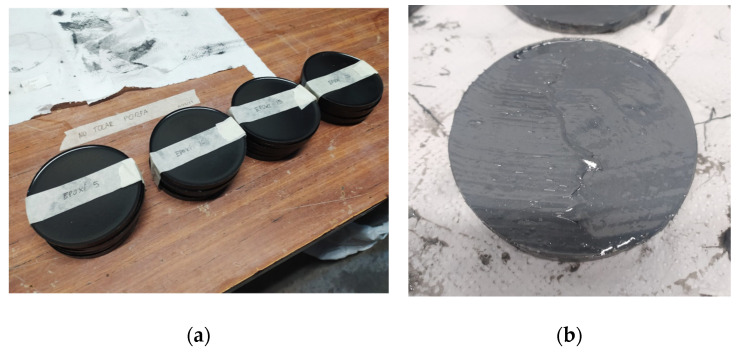
(**a**) Epoxy specimens cured at room temperature, demolded, and classified according to GNP concentration; (**b**) Polyurethane specimen leveled off in the mold, detail image.

Once the mixture was prepared, it was promptly poured into molds, as curing begins immediately. Sikadur has a pot life of 70 min at 20 °C, and Sikaforce, 155 min at 23 °C. The specimens were cured for at least 24 hours at room temperature before demolding. After curing at room temperature, the first electrical-conductivity test was performed on the specimens, prior to annealing at 120 °C for 120 min. Figure 1 shows examples of the (a) epoxy +5% volume GNP and (b) polyurethane +5% volume GNP composites.

**Figure 2:**
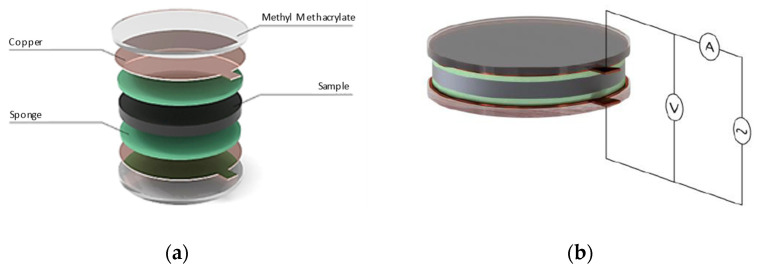
(**a**) Expanded assembly of the test setup adjacent to the copper electrodes. (**b**) Scheme of the electrical circuit used to take conductivity measurements.

To characterize sample conductivity, a two-point uniaxial method was used to calculate resistivity [5]. The specimen was placed between two electrodes, completing the circuit, and an alternating current was applied. The voltage between the electrodes was then measured. Measurements were taken within 2–3 s. The setup for the scheme is shown in Figure 2.

As shown, two sponges were added, one on each side of the specimen, each moistened with two pumps of hydroalcoholic gel to reduce contact resistance with the electrodes. The sponges were changed for each sample to maintain consistent conditions across tests.

Methyl methacrylate plates allowed the assembly to be clamped to ensure contact between all parts.

In terms of obtaining the conductivity value, resistivity is an intrinsic material property. Equation (1) shows the relationship between resistivity and resistance:

*ρ* = *k* · *R* (1)

Resistivity (*ρ*) is related to resistance (R) through a geometric factor (k), which depends on the specimen’s geometry and electrode distance, as shown in Equation (2):

(2) $$k=\frac{A}{L}$$

where A is the cross-sectional area perpendicular to the current direction, i.e., the area of the specimen in contact with the electrodes, and L is the thickness of the adhesive sample. Resistance (R) can be determined using Ohm’s law, as shown in Equation (3), where V represents the power source potential, and I is the current intensity measured with an ammeter:

(3) $$R=\frac{V}{I}$$

Finally, the value of electrical resistivity can be calculated. To obtain conductivity (*σ*) values, simply calculate the inverse of resistivity (*ρ*) using Equation (4):

(4) $$\sigma =\frac{1}{\rho }$$

The following instruments were used for the conductivity test setup: a PROMAX BF GBT-200-B generator as an AC power source, a PROMAX MD-200 digital multimeter as a voltmeter, and an ISO-TECH IDM 203 digital multimeter as an ammeter.

**Figure 3:**
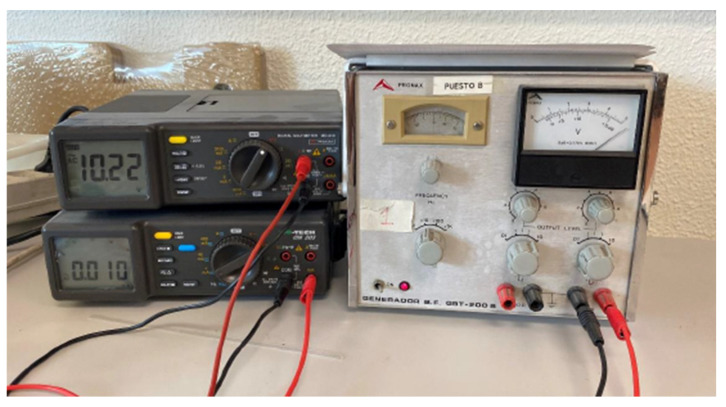
Voltmeter (**up-left**), ammeter (**down-left**), and alternating-current source (**right**).

The current source was set to approximately 10,000 Hz and 12 V, as AC voltage varies; voltage values were noted for each measurement (Figure 3).

As seen in Equation (2), knowledge of the sample’s geometry is essential, so the diameter and thickness of each specimen were measured with a digital caliper. Three diameter measurements and five thickness measurements were taken and averaged for each specimen.

After the test, all specimens were subjected to annealing at 120 °C for 120 min. Once this was completed and the samples cooled to room temperature, the conductivity test was repeated to compare the findings with the previous results.

**Figure 4:**
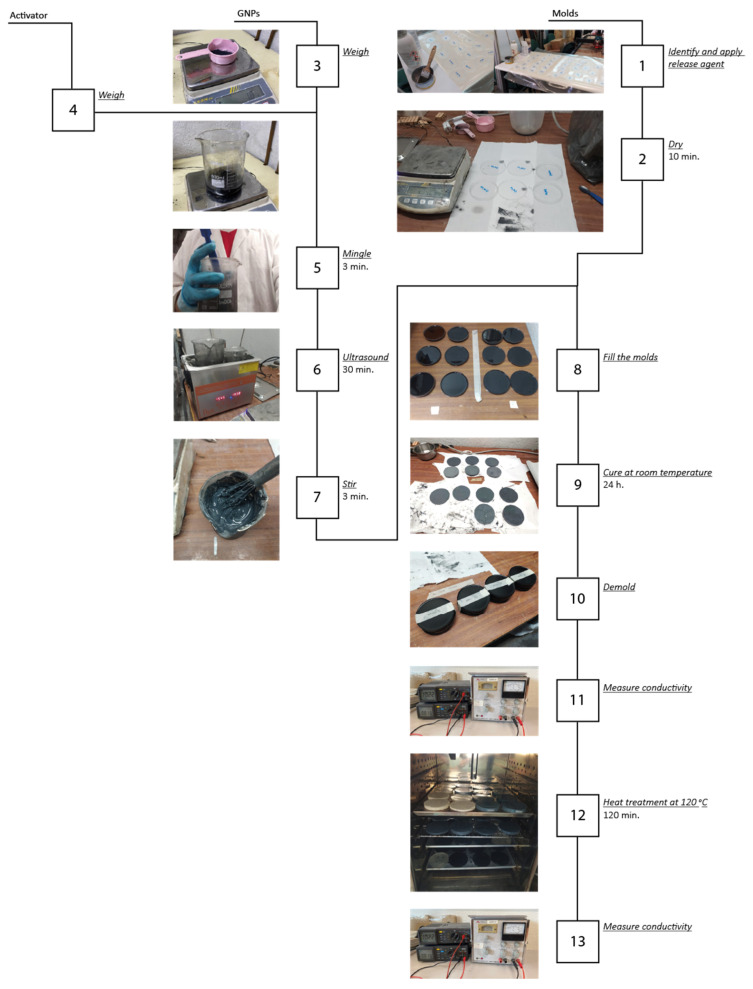
Experimental procedure.

In Figure 4, a scheme is shown summarizing the experimental procedure followed. It is worth noting that for each concentration, 6 samples were produced for each adhesive, that is, 12 samples in total for each GNP percentage. An exception was made for the 40% concentration, which was only tested using the epoxy-based adhesive, Sikadur 52.

## 3. Results

The volume percentages of filler concentration evaluated were 0, 5, 10, 15, 20, 25, and 40%. Note that specimens with a 40% GNP concentration were prepared only with the Sikadur epoxy resin adhesive, as the polyurethane adhesive was too viscous to handle at high GNP concentrations. Six specimens were measured for each percentage and adhesive type.

Table 3 shows the conductivity measurements for the specimens before (BC) and after curing (AC). In the case of Sikadur 52, some intensity and resistivity values deviate significantly within the same percentage (see Appendix A). Outliers were excluded, likely originating in defects or manufacturing errors, such as air bubbles or trapped moisture in the sample or differences in specimen volumes (all measurements are in Appendix A), as volume differences would affect the K factor.

### Conductivity

As can be observed, regarding Sikadur, the conductivity in certain percentages has increased significantly, particularly at 0% and 40%. However, other percentages, from 5% to 20%, have decreased. This may be due to the thermal and electrical aging of the specimens, as curing occurred after several weeks. Furthermore, the high curing temperature (120 °C) may have degraded the samples.

Concerning the conductivity measurements of the Sikaforce-422 L45 (polyurethane) specimens, in these cases, by leveling the specimens to the edge of the molds, all thickness measurements—and thus their associated K factors—were much more consistent, yielding more reliable results. However, there was still variability in the conductivity of each specimen, likely due to the uneven dispersion of GNPs within the polymer matrix.

For polyurethane specimens, after curing, conductivity increased across all percentages, although to a small extent. The only percentage that decreased was that of the 0% sample, i.e., pure polyurethane, which may have aged post-curing, leading to decreased conductivity. These results align with the work of Jorge Canales and colleagues [2], who applied the same curing conditions to polyurethane samples with reduced graphene oxide, suggesting that improvements in conductivity in this work would be more expected in polyurethane specimens.

**Figure 5:**
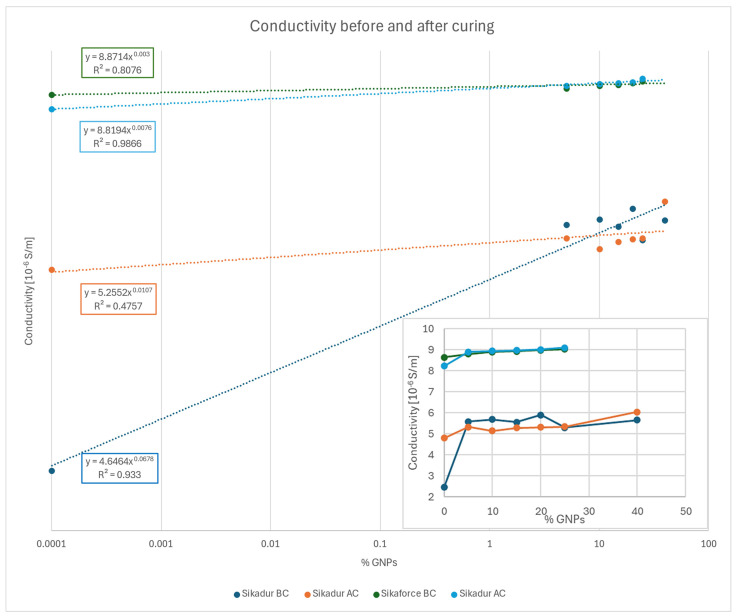
Sikadur and Sikaforce conductivity before (BC) and after (AC) curing, in both logarithmic and linear scales.

In Figure 5, the conductivity values from both adhesives, before and after curing, are represented in a logarithmic scale, including a power trend line to facilitate interpretation of the results. For both adhesives, conductivity increased as the quantity of GNPs rose. However, the slope for the Sikaforce adhesive is notably low, indicating very minor variations in values, even after curing. For Sikadur, the slope is significantly higher, especially before curing, resulting in greater conductivity growth with increases in filler.

In addition, in the bottom right corner of the graph, the data are presented on a linear scale.

As to the results before curing, both trend lines show high correlation coefficients, 0.81 and 0.93, which allow one to reliably accept the obtained results.

Regarding the conductivity in both adhesives after curing, both trend lines have a positive slope, indicating that conductivity increases with filler content. However, both slopes are low, as well as lower than the ones before curing, indicating a gradual increase. In this case, the correlation coefficient (R^2^) is very high for the Sikaforce adhesive (0.98) but significantly lower for the Sikadur adhesive (0.48), likely due to the final conductivity value of Sikadur, which deviates notably from the linear trend of the other points. The difference between this conductivity value and the immediately preceding one is 0.7 × 10^−6^ S/m, much higher than for the other specimens, which may suggest an approach to a magnitude change threshold. However, the difference is not significant enough to be considered the percolation threshold.

Concerning the Sikadur data, it is observed that conductivity before curing increases, with a slope approximately six times greater than after curing. Furthermore, the pre-cure trend line has an R^2^ coefficient of 0.93, demonstrating data reliability. In contrast, for cured adhesive, the coefficient is only 0.48, making predictions unreliable and indicating greater variability after curing. This is further reinforced by its mean standard deviation value, which is approximately four times higher than those exhibited by the polyurethane specimens before and after curing, demonstrating that the curing conditions are not optimal for the epoxy matrix, among other factors such as small variations in the dispersion of GNPs during sample preparation or differences in the viscosity of the mixture at high GNP concentrations. Material degradation during the curing process, particularly at elevated temperatures, could have influenced the stability of the electrical properties. Epoxy, in particular, is more sensitive to changes in curing conditions, which may have caused less predictable behavior regarding conductivity.

As for Sikaforce, once cured, the slope is more than twice that of the uncured adhesive. This could suggest that the curing parameters were more suitable for this type of matrix. Both R^2^ coefficients are high enough to validate the trend, especially for the cured adhesive. Notably, the conductivity values associated with specimens without filler decreased significantly post-curing, suggesting a potential negative effect of thermal aging on matrix conductivity.

In polyurethane specimens, a steady increase in conductivity is observed with higher GNP proportions and curing. For the epoxy adhesive, values show greater variability. As seen in the uncured conductivity tests, this may be due to the brief ultrasonic bath treatment, which affects the uniform distribution of the reinforcement in the matrix [1], especially in specimens with high GNP volume percentages, a factor which notably increases the mixture’s viscosity. The conductivity of the unreinforced adhesives increased in epoxy and decreased in polyurethane. This may indicate the impact of thermal aging on each resin type.

To verify the good dispersion within the matrices of the two polymers, the surface morphologies of the samples of GNPs and prepared adhesive (epoxy and polyurethane) were analyzed by a scanning electron microscope (SEM), model JEOL JSM 5600. The specimens were metalized with gold by sputtering in an argon atmosphere, and an electron acceleration voltage of 5 kV was used for the imaging.

**Figure 6:**
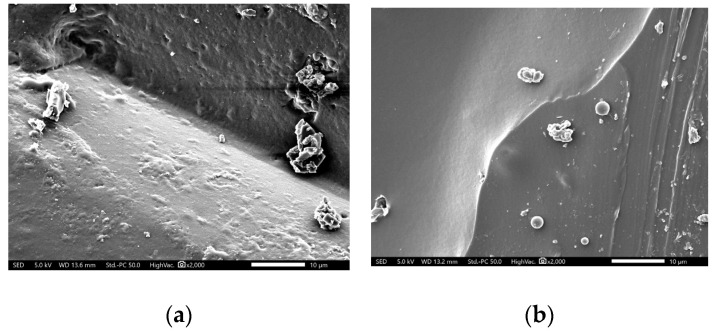
SEM micrographs of the (**a**) epoxy and (**b**) polyurethane samples (×2000).

Figure 6 shows examples of (a) epoxy +5% volume GNP and (b) polyurethane +5% volume GNP composites. Figure 6a shows areas of agglomeration that highlight the dispersion difficulties encountered and may explain the low conductivity achieved compared to values in the literature, as well as the variability in the results. Meanwhile, in Figure 6b, the GNPs appear to be well distributed within the polymer matrix, and without evidence of agglomerations, supporting the concept of the availability of the polyurethane monomers to the edges of the GNPs. GNPs appear to be homogeneously distributed in the matrix and they are not agglomerated; additionally, a good interfacial interaction between the GNPs and the polyurethane matrix can be noticed.

It is worth mentioning that M.E. Demir et al. [6], in GNP-epoxy composites, found that higher reinforcement ratios significantly improved the mechanical strength and reduced wear, up to an optimal point after which GNP agglomeration occurs, which limits performance. In addition, S. Akpinar and his team [7] analyzed how the combination of surface roughness and nanostructures of the adhesive affects the strength of bonded joints. The results show that surfaces with optimal roughness and well-designed nanostructures significantly improve adhesion strength by increasing the effective contact area and mechanical interactions, stressing the importance of this nanostructure.

## 4. Discussion

This study has provided a clear understanding of the electrical behavior of commercial adhesives reinforced with GNPs, focusing on identifying the processing conditions that optimize their conductivity and adaptability for industrial applications. The key findings are discussed below based on the three specific objectives outlined.

As to the impact of ultrasonic time on GNP dispersion and exfoliation, the results indicate that the limited ultrasonic time (30 min) affects the homogeneity of GNP dispersion, particularly at high GNP concentrations. This is evident in epoxy resin samples, which exhibited greater variability in conductivity compared to polyurethane. While longer ultrasonic times could improve dispersion, previous studies suggest that this might lead to GNP fragmentation and a reduction in specific surface area, ultimately compromising final conductivity. Therefore, it is crucial to optimize this parameter by balancing homogeneity with the preservation of filler structure.

As to the influence of additional thermal treatment on electrical conductivity, thermal curing at 120 °C for 120 min proved to be a critical factor in enhancing the conductivity of both matrices. In polyurethane samples, conductivity consistently increased with GNP concentration after thermal treatment, suggesting that this type of matrix is more compatible with such conditions. In contrast, epoxy samples displayed greater variability, likely due to manufacturing defects such as air bubbles or insufficient filler dispersion, which are exacerbated during curing. This finding underscores the importance of adjusting curing parameters according to the type of matrix to maximize electrical performance.

As to the comparison of performance between epoxy and polyurethane matrices, the results show that polyurethane matrices, under the same processing conditions, exhibit higher and more consistent conductivity than epoxy matrices. This can be attributed to the intrinsic properties of polyurethane, such as greater flexibility and lower viscosity, which facilitate a more uniform dispersion of GNPs. Conversely, epoxy resin, while allowing higher GNP concentrations (up to 40% by volume), showed limitations in mixture homogeneity due to increased viscosity at higher filler concentrations. These results suggest that polyurethane is a more viable option for industrial applications requiring stable electrical conductivity and simpler processing methods.

Although many studies place the percolation threshold above 3% by mass (approximately 40% by volume), samples with GNP percentages beyond these values could not be produced. No GNP concentration was found that led to an order-of-magnitude increase in conductivity (percolation threshold). This could be an inherent characteristic of the GNP systems used, as GNPs have a different geometry and behavior compared to other conductive fillers, such as carbon nanotubes or reduced graphene oxide. In comparison to these other systems, GNPs, due to their larger size and tendency to stack, may require higher concentrations or specific processing conditions to achieve percolation. Future studies plan to investigate this phenomenon further by using different types of fillers or more advanced dispersion techniques to explore their effect on the percolation threshold and the conductive properties of the system.

Using the uniaxial two-point method for conductivity measurements considers the specimen volume, allowing for differences in specimen thickness. However, manufacturing defects such as bubbles, small cracks, or trapped moisture may have affected conductivity variability. A promising option to minimize these defects would be to press the mixture after pouring it into the mold or apply vibrations to expel trapped air while the mixture is fresh.

Conductivity values were obtained for all percentages, although lower in magnitude than reported in the literature. The highest conductivity values obtained were 6.05 × 10^−6^ S/m for the epoxy matrix (40% GNP) and 9.1 × 10^−6^ S/m for the polyurethane matrix (25% GNP). In contrast, S. Prolongo obtained 0.004 S/m [4] with a GNP–epoxy composite, and Jorge Canales obtained 0.003 S/cm [2] with polyurethane and reduced graphene oxide. These results are influenced by various factors, including reinforcement dispersion, filler type, matrix material, curing temperature and time, etc. However, comparing results from this study with those from other articles is challenging [1,2,3,4,8] as the latter used different graphene derivatives, polymers, specimen manufacturing methods, and conductivity measurement techniques. For instance, Canales [2], used reduced graphene oxide (rGO) as filler, which has superior electrical properties due to the higher specific surface area and its more conductive structure, compared to the GNPs used in this study. While GNPs have good conductivity, due to their geometry, they have a greater tendency to stack, which can hinder the formation of a continuous conductive network at lower concentrations.

The present study offers several significant contributions to the field of conductive adhesives which enhance the understanding and practical applications of these materials. The direct comparison between epoxy and polyurethane matrices reinforced with GNPs, conducted under identical experimental conditions, provides new insights into the electrical properties of these materials. Furthermore, the study evaluates a wide range of filler concentrations (0–40% by volume for epoxy and 0–25% for polyurethane), enabling a more detailed analysis of the practical limitations that arise due to increasing GNP content, particularly in terms of material viscosity. This focus on commercial adhesives such as Sikadur and Sikaforce adds a unique industrial perspective, one which is often overlooked in previous studies. Another important aspect of this work is the evaluation of the thermal curing treatment (120 °C for 120 min), which explores its impact on the conductivity of the composites. While high-temperature curing has been explored before, this study distinguishes itself by comparing its effects across two different matrix types, revealing how they affect each matrix differently. Lastly, the study addresses the crucial challenge of GNP dispersion in adhesive systems.

Based on this study’s findings, several directions for further investigation are proposed to enhance and fully exploit the potential of GNPs as filler for improved conductivity values.

First, the effects of varying ultrasonic bath times for a single GNP concentration could be explored to optimize filler dispersion and achieve a homogeneous mixture with minimal GNP breakage. This would confirm claims in the literature that excessive ultrasonic time decreases composite conductivity due to GNP fragmentation and reduced specific area. Additionally, the effects of bath temperature on conductivity levels could be assessed.

Regular conductivity measurements subsequent to specimen manufacture would help evaluate the effect of time on conductivity levels, both before and after specimen curing.

Another necessary avenue of investigation would be to examine curing parameters. For instance, using different time and temperature settings for the same GNP concentration and matrix could identify conditions that optimize conductivity. Moreover, evaluating conductivity evolution after multiple annealing cycles, rather than a single cycle, may improve conductivity, as the literature suggests that a second cure often yields notable conductivity gains.

In general, optimizing GNP usage should be the main objective, as the high cost of conductive fillers and composite manufacturing limits their widespread use.

Moreover, hardness tests, flexural tests, or even impermeability tests would be of interest. This would allow for the analysis of the impacts of filler addition on the composite material’s mechanical properties (as other colleagues in the field have shown [9,10]), as these materials are industrially applied and certain properties beyond electrical conductivity must be ensured. S.K. Yadav et al. [11] discuss the use of functionalized GNPs in polyurethane matrices. The results show that the incorporation of these GNPs significantly improves the tensile strength, stiffness, and thermal conductivity of the material, which is attributed to the uniform dispersion and the chemical interactions between the GNP and the polyurethane matrix. On the other hand, Z.M. Jia et al. [12] investigated the effects of water immersion on the shear strength of GNP-reinforced epoxy adhesives. The results reveal that the incorporation of GNP enhances the initial shear strength of the adhesive, although prolonged exposure to water reduces this improvement, due to chemical and physical degradation. Using Pearson correlation matrices and heatmaps to interpret the data, N.Z. Khalil et al. [13] show that GNPs significantly enhance the stiffness, strength, and thermal stability of epoxy composites.

## 5. Conclusions

This study provides valuable insights into the electrical-conductivity behavior of epoxy and polyurethane matrices reinforced with GNPs. Conductivity values were achieved for all concentrations, though they were lower in magnitude compared to those reported in the literature. It was observed that as the filler content increased, conductivity increased for both adhesives. The highest conductivity values recorded were 6.05 × 10 ^−6^ S/m for the epoxy matrix (40% GNP) and 9.1 × 10 ^−6^ S/m for the polyurethane matrix (25% GNP).

The results reveal that polyurethane exhibits higher and more consistent conductivity improvements when tested with GNP content, compared to epoxy. Variability in epoxy samples, as observed in the R2 coefficient of the associated trend line after thermal treatment (0.4757) and in the related standard deviation values, can be attributed to intrinsic material properties, as well as inadequate dispersion and defects like air bubbles, suggesting a need for refined processing techniques. Moreover, while no percolation threshold was noticed, a shift of 0.7 × 10 ^−6^ S/m was observed between the maximum conductivity value obtained for the epoxy samples (corresponding to a 40% GNP concentration) after curing and the value preceding it. This shift is seven times greater than the maximum increase observed in the polyurethane samples. This could be an indication of approaching the percolation threshold. The study underscores the potential of optimizing processing conditions, such as ultrasonic bath duration and curing cycles, to enhance conductivity.

Both the physical and chemical properties of the polyurethane and epoxy matrices play crucial roles in the dispersion of GNPs and the electrical behavior of the composites. Polyurethane, with its higher flexibility, promotes a more homogeneous dispersion of the GNPs, facilitating the formation of more efficient conductive networks and reducing variability in conductivity measurements. In contrast, epoxy, while allowing for higher concentrations of GNPs, hinders uniform dispersion, especially at higher filler concentrations, thus increasing variability in conductivity. Additionally, differences in the chemistry of the resins, such as their behavior during curing and interaction with the GNPs, also contribute to the variability observed in the electrical properties of the composites.

By comparing these findings with previous studies, this article identifies key factors influencing conductivity, including filler type, matrix material, and processing methods. The data reinforce the necessity of tailoring manufacturing parameters to the specific adhesive and filler combination to maximize performance. Mechanical testing is also recommended to evaluate how GNP addition impacts properties such as hardness and flexibility, ensuring the suitability of these composites for industrial applications.

GNPs demonstrate significant potential as a conductive filler [14] due to their cost-effectiveness, versatility, and compatibility with polymer matrices. Coupled with the widespread use of epoxy and polyurethane adhesives in industries such as construction, automotive, and electronics, these materials present a transformative opportunity to develop lightweight, conductive adhesives. Such composites could replace conventional wiring and electrical connections, offering innovative solutions for next-generation technologies across multiple sectors.

This study makes a significant contribution by offering important findings on the performance of epoxy and polyurethane matrices reinforced with GNPs, comparing both matrices under the same experimental conditions and emphasizing their distinct behaviors. The evaluation of a wide spectrum of filler concentrations and with respect to the thermal curing treatment offers a deeper understanding of their impacts on conductivity and material viscosity, particularly in commercial adhesives like Sikadur and Sikaforce. Additionally, the study emphasizes the importance of optimizing GNP dispersion in adhesive systems, contributing to the development of more efficient and commercially viable conductive adhesives.

## Figures and Tables

**Table 1:** Sikadur 52 and SikaForce-422 L45: physical properties.

**Sikadur 52**	**Comp. A**	**Comp. B**	**Mixture**
Mixture proportion (in volume and weight)	2	1	
Density at 20 °C [g/cm^3^]	1.1	1	1.1
Viscosity at 20 °C [Pa·s]			0.33
Lifespan at 20 °C of 1000 g [min]			70
**SikaForce-422 L45**	**Polyol**	**Isocyanate**	**Mixture**
Mixture proportion (in weight)	100	25	
Mixture proportion (in volume)	100	19	
Viscosity at 23 °C [mPa·s]	115,000	300	45,000
Density (before curing) [g/cm^3^]	1.60	1.23	1.53

**Table 2:** GNPs’ properties.

Lateral size (LD50) [μm]	7
Average thickness [nm]	3
Oxygen content (XPS)	<1%
BET [m^2^/g]	70
Average number of layers: multilayer	5–10

**Table 3:** Conductivity before and after curing.

	Conductivity [S/m]					
**GNP Volume [%]**						
0	2.45031 × 10^−6^	4.8048 × 10^−6^	96	8.6456 × 10^−6^	8.2306 × 10^−6^	−5
5	5.5927 × 10^−6^	5.33131 × 10^−6^	−5	8.8115 × 10^−6^	8.9032 × 10^−6^	1
10	5.6875 × 10^−6^	5.1471 × 10^−6^	−10	8.9118 × 10^−6^	8.9454 × 10^−6^	0
15	5.557 × 10^−6^	5.2811 × 10^−6^	−5	8.931 × 10^−6^	8.9831 × 10^−6^	1
20	5.8982 × 10^−6^	5.3225 × 10^−6^	−10	8.9964 × 10^−6^	9.0192 × 10^−6^	0
25	5.30402 × 10^−6^	5.34 × 10^−6^	1	9.0335 × 10^−6^	9.1076 × 10^−6^	1
40	5.6638 × 10^−6^	6.0478 × 10^−6^	7	-	-	−5
	Sikadur 52 BC	Sikadur 52 AC	Sikadur Variation [%]	Sikaforce-422 L45 BC	Sikaforce-422 L45 AC	Sikaforce Variation [%]
**Mean standard deviation**	0.015	0.137	-	0.030	0.036	-

**Figure A1:**
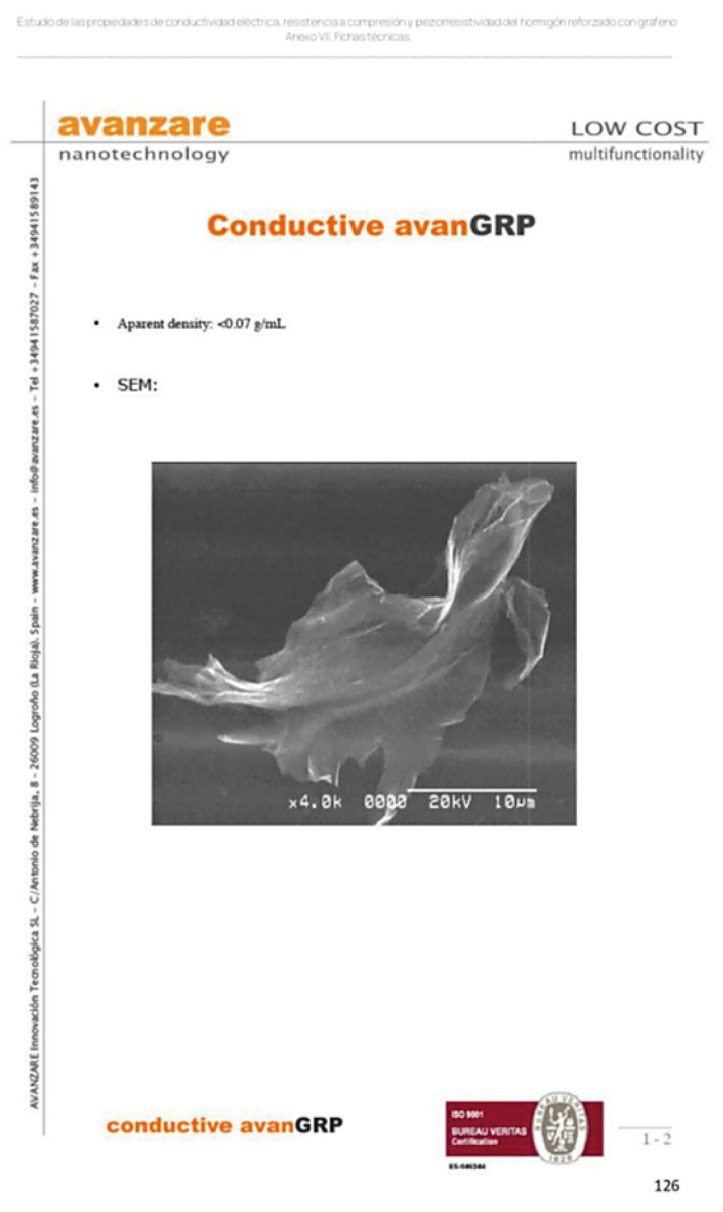
The av-PLAT-7 technical sheet, p. 1.

**Figure A2:**
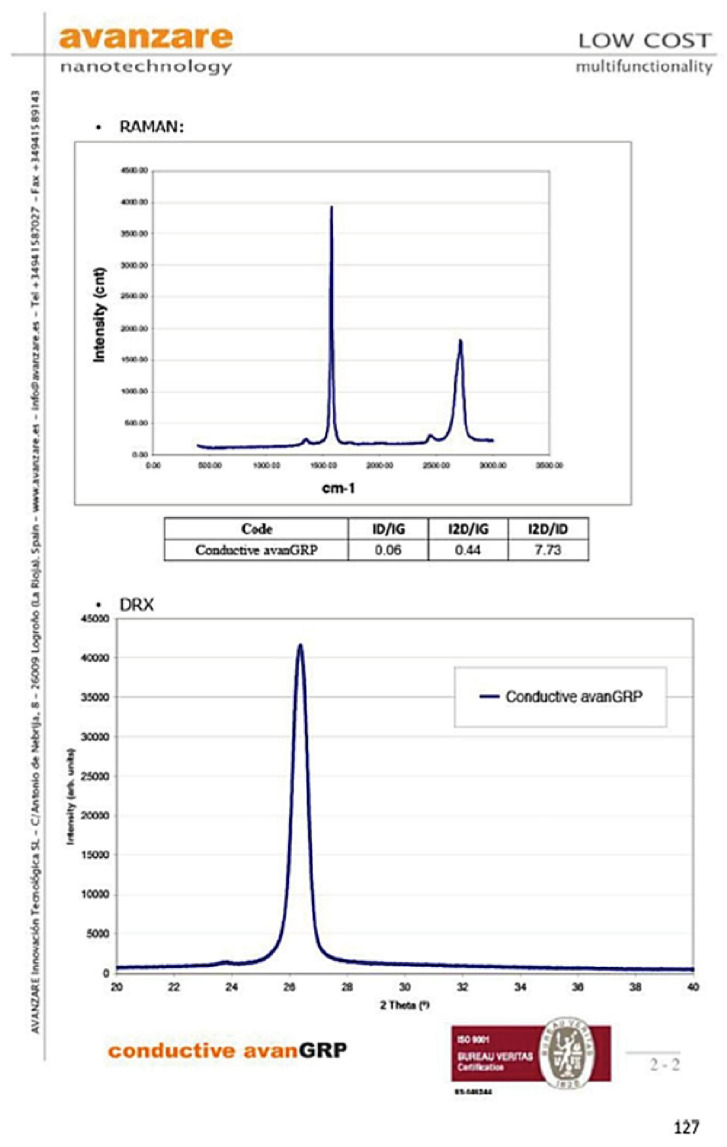
The av-PLAT-7 technical sheet, p. 2.

## Data Availability

Complete data will be made available on request.

## Appendix A. Conductivity Measures

**Table A1:** Sikadur conductivity before curing.

% GNP	Specimen	D _mean_ [mm]	T _mean_ [mm]	Voltage [V]	Amperage [μA]	R [Ω]	S [mm^2^]	k [mm]	Resistivity [Ωmm]	Conductivity [S/m]
0	1	89.64	3.848	11.97	32.4	0.3694	6310.9331	1640.0554	605.9093	1.6504 × 10^−6^
	2	89.9	3.846	12.02	36.1	0.3330	6347.5958	1650.4409	549.5374	1.8197 × 10^−6^
	3	89.9066667	4.008	11.92	45.7	0.2608	6348.5373	1583.9664	413.1483	2.4204 × 10^−6^
	4	86.2833333	6.212	12.07	58.6	0.2060	5847.1429	941.2658	193.8750	5.158 × 10^−6^
	5	86.3166667	6.288	12.02	36.2	0.3320	5851.6616	930.6078	309.0029	3.2362 × 10^−6^
	6	86.25	6.154	12	35.6	0.3371	5842.6260	949.4030	320.0235	3.1248 × 10^−6^
5	1	89.6166667	3.99	12.06	87.7	0.1375	6307.6481	1580.8642	217.3914	4.6 × 10^−6^
	2	89.72	3.388	12.05	92	0.1310	6322.2026	1866.0574	244.4130	4.0914 × 10^−6^
	3	89.77	4.26	12.03	87.4	0.1376	6329.2512	1485.7397	204.5017	4.8899 × 10^−6^
	4	86.2566667	5.838	12.02	81.4	0.1477	5843.5293	1000.9471	147.8057	6.7656 × 10^−6^
	5	86.14	5.112	12.01	85.1	0.1411	5827.7326	1140.0103	160.8875	6.2155 × 10^−6^
	6	86.2733333	6.176	12.01	79.5	0.1511	5845.7877	946.5330	142.9920	6.9934 × 10^−6^
10	1	89.7833333	4.344	11.98	87	0.1377	6331.1315	1457.4428	200.6915	4.9828 × 10^−6^
	2	89.75	4.056	11.98	89.6	0.1337	6326.4313	1559.7710	208.5497	4.795 × 10^−6^
	3	89.8066667	4.236	11.96	92.9	0.1287	6334.4226	1495.3783	192.5159	5.1944 × 10^−6^
	4	86.0733333	5.072	12.02	86.3	0.1393	5818.7155	1147.2231	159.7870	6.2583 × 10^−6^
	5	86.0866667	4.894	12.01	92.4	0.1300	5820.5184	1189.3172	154.5855	6.4689 × 10^−6^
	6	86.1566667	5.264	12	85.4	0.1405	5829.9880	1107.5205	155.6235	6.4258 × 10^−6^
15	1	89.7466667	3.464	11.98	98.5	0.1216	6325.9614	1826.2013	222.1106	4.5023 × 10^−6^
	2	89.69	4.064	11.97	92.4	0.1295	6317.9754	1554.6199	201.3939	4.9654 × 10^−6^
	3	89.7133333	4.496	11.97	89.1	0.1343	6321.2631	1405.9749	188.8835	5.2943 × 10^−6^
	4	85.96	3.928	11.97	92.9	0.1288	5803.4025	1477.4446	190.3661	5.253 × 10^−6^
	5	86.05	4.784	12.05	89	0.1354	5815.5612	1215.6273	164.5877	6.0758 × 10^−6^
	6	86.13	6.092	12.04	83.5	0.1442	5826.3796	956.3985	137.9046	7.2514 × 10^−6^
20	1	89.7733333	4.04	12.02	101.1	0.1189	6329.7212	1566.7627	186.2758	5.3684 × 10^−6^
	2	89.69	3.446	12.01	103.6	0.1159	6317.9754	1833.4229	212.5426	4.7049 × 10^−6^
	3	89.7733333	3.78	12	102.2	0.1174	6329.7212	1674.5294	196.6179	5.086 × 10^−6^
	4	86.2333333	5.868	11.98	93.2	0.1285	5840.3682	995.2911	127.9355	7.8164 × 10^−6^
	5	86.01	3.988	11.98	100.5	0.1192	5810.1558	1456.9097	173.6694	5.7581 × 10^−6^
	6	86.1733333	4.618	11.98	100.7	0.1190	5832.2438	1262.9371	150.2481	6.6557 × 10^−6^
25	1	89.7966667	3.27	11.98	90.8	0.1319	6333.0120	1936.7009	255.5251	3.9135 × 10^−6^
	2	89.9	3.686	11.96	97	0.1233	6347.5958	1722.0824	212.3310	4.7096 × 10^−6^
	3	89.9	3.58	11.96	92.2	0.1297	6347.5958	1773.0715	229.9993	4.3478 × 10^−6^
	4	86.2	3.89	11.95	98.4	0.1214	5835.8539	1500.2195	182.1913	5.4887 × 10^−6^
	5	86.1433333	4.574	11.95	94.2	0.1269	5828.1836	1274.1984	161.6419	6.1865 × 10^−6^
	6	86.3866667	4.166	11.95	97.3	0.1228	5861.1565	1406.9027	172.7902	5.7874 × 10^−6^
40	1	89.9466667	2.166	11.95	176.8	0.0676	6354.1875	2933.6046	198.2838	5.0433 × 10^−6^
	2	89.8333333	3.062	11.94	137.2	0.0870	6338.1850	2069.9494	180.1399	5.5512 × 10^−6^
	3	89.8966667	3.66	11.92	127.4	0.0936	6347.1251	1734.1872	162.2568	6.1631 × 10^−6^
	4	89.85	3.622	11.92	126.3	0.0944	6340.5370	1750.5624	165.2154	6.0527 × 10^−6^
	5	89.8766667	2.8	11.9	146	0.0815	6344.3012	2265.8219	184.6800	5.4148 × 10^−6^
	6	89.8966667	2.924	11.89	148.6	0.0800	6347.1251	2170.6994	173.6852	5.7575 × 10^−6^

**Table A2:** Sikadur conductivity once cured.

% GNP	Specimen	D _mean_ [mm]	T _mean_ [mm]	Voltage [V]	Amperage [μA]	R [Ω]	S [mm^2^]	k [mm]	Resistivity [Ωmm]	Conductivity [S/m]
0	1	89.64	3.848	11.9	78.9	0.1508	6310.9331	1640.0554	247.3594	4.0427 × 10^−6^
	2	89.9	3.846	11.86	78.4	0.1513	6347.5958	1650.4409	249.6713	4.00527 × 10^−6^
	3	89.9066667	4.008	11.83	77.7	0.1523	6348.5373	1583.9664	241.1624	4.14658 × 10^−6^
	4	86.2833333	6.212	11.79	60.3	0.1955	5847.1429	941.2658	184.0385	5.43364 × 10^−6^
	5	86.3166667	6.288	11.77	62.3	0.1889	5851.6616	930.6078	175.8147	5.68781 × 10^−6^
	6	86.25	6.154	11.75	61.5	0.1911	5842.6260	949.4030	181.3900	5.51298 × 10^−6^
5	1	89.6166667	3.99	12.07	79.6	0.1516	6307.6481	1580.8642	239.7114	4.17168 × 10^−6^
	2	89.72	3.388	12.06	84.3	0.1431	6322.2026	1866.0574	266.9591	3.74589 × 10^−6^
	3	89.77	4.26	12.05	80.7	0.1493	6329.2512	1485.7397	221.8484	4.50758 × 10^−6^
	4	86.2566667	5.838	12.04	74.4	0.1618	5843.5293	1000.9471	161.9812	6.17355 × 10^−6^
	5	86.14	5.112	12.03	77.9	0.1544	5827.7326	1140.0103	176.0504	5.68019 × 10^−6^
	6	86.2733333	6.176	12.03	73.7	0.1632	5845.7877	946.5330	154.5019	6.47241 × 10^−6^
10	1	89.7833333	4.344	12.01	78.6	0.1528	6331.1315	1457.4428	222.6958	4.49043 × 10^−6^
	2	89.75	4.056	12	81.4	0.1474	6326.4313	1559.7710	229.9417	4.34893 × 10^−6^
	3	89.8066667	4.236	12	80.67	0.1488	6334.4226	1495.3783	222.4438	4.49552 × 10^−6^
	4	86.0733333	5.072	11.99	78.5	0.1527	5818.7155	1147.2231	175.2255	5.70693 × 10^−6^
	5	86.0866667	4.894	11.98	84.3	0.1421	5820.5184	1189.3172	169.0157	5.91661 × 10^−6^
	6	86.1566667	5.264	11.98	78.6	0.1524	5829.9880	1107.5205	168.8053	5.92399 × 10^−6^
15	1	89.7466667	3.464	11.96	84.9	0.1409	6325.9614	1826.2013	257.2599	3.88712 × 10^−6^
	2	89.69	4.064	11.96	83.1	0.1439	6317.9754	1554.6199	223.7455	4.46936 × 10^−6^
	3	89.7133333	4.496	11.96	81.4	0.1469	6321.2631	1405.9749	206.5781	4.84078 × 10^−6^
	4	85.96	3.928	11.94	85.6	0.1395	5803.4025	1477.4446	206.0828	4.85242 × 10^−6^
	5	86.05	4.784	11.93	80.7	0.1478	5815.5612	1215.6273	179.7080	5.56458 × 10^−6^
	6	86.13	6.092	11.93	76.2	0.1566	5826.3796	956.3985	149.7354	6.67845 × 10^−6^
20	1	89.7733333	4.04	11.92	90.2	0.1322	6329.7212	1566.7627	207.0489	4.82978 × 10^−6^
	2	89.69	3.446	11.91	91.2	0.1306	6317.9754	1833.4229	239.4306	4.17658 × 10^−6^
	3	89.7733333	3.78	11.91	89.6	0.1329	6329.7212	1674.5294	222.5853	4.49266 × 10^−6^
	4	86.2333333	5.868	11.92	82.3	0.1448	5840.3682	995.2911	144.1539	6.93703 × 10^−6^
	5	86.01	3.988	11.91	95.7	0.1245	5810.1558	1456.9097	181.3145	5.51528 × 10^−6^
	6	86.1733333	4.618	11.91	90	0.1323	5832.2438	1262.9371	167.1287	5.98341 × 10^−6^
25	1	89.7966667	3.27	11.91	60.2	0.1978	6333.0120	1936.7009	383.1579	2.60989 × 10^−6^
	2	89.9	3.686	11.9	98.2	0.1212	6347.5958	1722.0824	208.6841	4.79193 × 10^−6^
	3	89.9	3.58	11.9	97.6	0.1219	6347.5958	1773.0715	216.1839	4.62569 × 10^−6^
	4	86.2	3.89	11.89	95.3	0.1248	5835.8539	1500.2195	187.1732	5.34264 × 10^−6^
	5	86.1433333	4.574	11.89	94.6	0.1257	5828.1836	1274.1984	160.1503	6.24413 × 10^−6^
	6	86.3866667	4.166	11.88	95.2	0.1248	5861.1565	1406.9027	175.5673	5.69582 × 10^−6^
40	1	89.9466667	2.166	12.02	193.1	0.0622	6354.1875	2933.6046	182.6097	5.47616 × 10^−6^
	2	89.8333333	3.062	12.03	146.1	0.0823	6338.1850	2069.9494	170.4414	5.86712 × 10^−6^
	3	89.8966667	3.66	12.04	131.3	0.0917	6347.1251	1734.1872	159.0222	6.28843 × 10^−6^
	4	89.85	3.622	12.03	135.3	0.0889	6340.5370	1750.5624	155.6487	6.42473 × 10^−6^
	5	89.8766667	2.8	12	167	0.0719	6344.3012	2265.8219	162.8135	6.142 × 10^−6^
	6	89.8966667	2.924	12	158.6	0.0757	6347.1251	2170.6994	164.2396	6.08867 × 10^−6^

**Table A3:** Sikaforce conductivity before curing.

% GNP	Specimen	D _mean_ [mm]	T _mean_ [mm]	Voltage [V]	Amperage [μA]	R [Ω]	S [mm^2^]	k [mm]	Resistivity [Ωmm]	Conductivity [S/m]
0	1	86.7	10.652	12.05	71.4	0.1688	5903.7516	554.2388	93.5375	1.0691 × 10^−5^
	2	86.4266667	10.502	11.93	71.2	0.1676	5866.5855	558.6160	93.5996	1.0684 × 10^−5^
	3	86.5233333	10.56	12.14	72.6	0.1672	5879.7162	556.7913	93.1053	1.0741 × 10^−5^
	4	90.03	5.946	12.09	84	0.1439	6365.9670	1070.6302	154.0943	6.4895 × 10^−6^
	5	89.9833333	5.654	12.06	88.4	0.1364	6359.3691	1124.7558	153.4452	6.517 × 10^−6^
	6	89.9033333	5.952	12.04	86.7	0.1389	6348.0665	1066.5434	148.1105	6.7517 × 10^−6^
5	1	86.1466667	10.57	11.97	72.3	0.1656	5828.6347	551.4319	91.2951	1.0953 × 10^−5^
	2	86.3266667	10.646	11.95	72.4	0.1651	5853.0175	549.7856	90.7450	1.102 × 10^−5^
	3	86.42	10.654	11.94	72.4	0.1649	5865.6805	550.5613	90.7970	1.1014 × 10^−5^
	4	90	5.866	11.92	82.7	0.1441	6361.7251	1084.5082	156.3161	6.3973 × 10^−6^
	5	89.98	6.06	12.06	87.2	0.1383	6358.8980	1049.3231	145.1243	6.8906 × 10^−6^
	6	89.7666667	5.932	12.04	84.7	0.1421	6328.7812	1066.8883	151.6568	6.5938 × 10^−6^
10	1	86.3833333	10.604	12.01	74.4	0.1614	5860.7041	552.6881	89.2175	1.1209 × 10^−5^
	2	86.38	10.654	12	72.5	0.1655	5860.2519	550.0518	91.0431	1.0984 × 10^−5^
	3	86.4266667	10.408	11.99	73.8	0.1625	5866.5855	563.6612	91.5758	1.092 × 10^−5^
	4	89.9233333	6.018	11.98	85.1	0.1408	6350.8912	1055.3159	148.5627	6.7312 × 10^−6^
	5	89.7933333	6.158	11.97	82.5	0.1451	6332.5419	1028.3439	149.2034	6.7023 × 10^−6^
	6	89.8033333	6.172	11.96	85	0.1407	6333.9524	1026.2399	144.3980	6.9253 × 10^−6^
15	1	86.2633333	10.614	11.94	71.9	0.1661	5844.4326	550.6343	91.4405	1.0936 × 10^−5^
	2	86.33	10.614	11.94	72.2	0.1654	5853.4695	551.4857	91.2014	1.0965 × 10^−5^
	3	86.4533333	10.646	12.05	72.5	0.1662	5870.2063	551.4002	91.6465	1.0911 × 10^−5^
	4	89.89	6.168	12.04	82.9	0.1452	6346.1837	1028.8884	149.4308	6.6921 × 10^−6^
	5	89.8833333	6.3	12.03	85.2	0.1412	6345.2425	1007.1813	142.2112	7.0318 × 10^−6^
	6	89.9066667	6.364	12.03	84.6	0.1422	6348.5373	997.5703	141.8531	7.0495 × 10^−6^
20	1	86.2833333	10.288	12.01	73.6	0.1632	5847.1429	568.3459	92.7423	1.0783 × 10^−5^
	2	86.2233333	10.09	12.01	78.6	0.1528	5839.0138	578.6931	88.4237	1.1309 × 10^−5^
	3	86.2733333	10.67	12	71.3	0.1683	5845.7877	547.8714	92.2084	1.0845 × 10^−5^
	4	89.9733333	6.226	11.99	83.8	0.1431	6357.9558	1021.1943	146.1112	6.8441 × 10^−6^
	5	89.9366667	6.154	11.98	87.8	0.1364	6352.7747	1032.3001	140.8537	7.0996 × 10^−6^
	6	89.7966667	6.388	11.98	84.3	0.1421	6333.0120	991.3920	140.8882	7.0978 × 10^−6^
25	1	86.2766667	9.91	11.97	77.2	0.1551	5846.2394	589.9333	91.4702	1.0933 × 10^−5^
	2	86.27	10.474	11.97	77.4	0.1547	5845.3360	558.0806	86.3078	1.1586 × 10^−5^
	3	86.2733333	9.666	11.97	79.3	0.1509	5845.7877	604.7784	91.2887	1.0954 × 10^−5^
	4	89.8866667	6.034	11.97	94.5	0.1267	6345.7131	1051.6594	133.2102	7.5069 × 10^−6^
	5	89.9033333	5.832	11.96	88.1	0.1358	6348.0665	1088.4888	147.7676	6.7674 × 10^−6^
	6	89.89	5.676	11.96	86.3	0.1386	6346.1837	1118.0732	154.9497	6.4537 × 10^−6^

**Table A4:** Sikaforce conductivity once cured.

% GNP	Specimen	D _mean_ [mm]	T _mean_ [mm]	Voltage [V]	Amperage [μA]	R [Ω]	S [mm^2^]	k [mm]	Resistivity [Ωmm]	Conductivity [S/m]
0	1	86.7	10.652	12.06	63.8	0.1890	5903.7516	554.2388	104.7668	9.545 × 10^−6^
	2	86.4266667	10.502	12.05	64.3	0.1874	5866.5855	558.6160	104.6862	9.5524 × 10^−6^
	3	86.5233333	10.56	12.05	66.5	0.1812	5879.7162	556.7913	100.8923	9.9116 × 10^−6^
	4	90.03	5.946	12.05	88.6	0.1360	6365.9670	1070.6302	145.6105	6.8676 × 10^−6^
	5	89.9833333	5.654	12.05	90.5	0.1331	6359.3691	1124.7558	149.7603	6.6773 × 10^−6^
	6	89.9033333	5.952	12.04	87.7	0.1373	6348.0665	1066.5434	146.4217	6.8296 × 10^−6^
5	1	86.1466667	10.57	12.03	73.1	0.1646	5828.6347	551.4319	90.7486	1.0748 × 10^−5^
	2	86.3266667	10.646	12.04	72.1	0.1670	5853.0175	549.7856	91.8089	1.059 × 10^−5^
	3	86.42	10.654	12.04	73.2	0.1645	5865.6805	550.5613	90.5568	1.0605 × 10^−5^
	4	90	5.866	12.03	86.5	0.1391	6361.7251	1084.5082	150.8281	6.6301 × 10^−6^
	5	89.98	6.06	12.05	87.2	0.1382	6358.8980	1049.3231	145.0039	6.5247 × 10^−6^
	6	89.7666667	5.932	12.05	89.2	0.1351	6328.7812	1066.8883	144.1256	6.4639 × 10^−6^
10	1	86.3833333	10.604	12.03	73	0.1648	5860.7041	552.6881	91.0800	1.0573 × 10^−5^
	2	86.38	10.654	12.03	72.1	0.1669	5860.2519	550.0518	91.7770	1.0564 × 10^−5^
	3	86.4266667	10.408	12.03	71.4	0.1685	5866.5855	563.6612	94.9698	1.053 × 10^−5^
	4	89.9233333	6.018	12.03	88	0.1367	6350.8912	1055.3159	144.2665	6.522 × 10^−6^
	5	89.7933333	6.158	12.02	88.3	0.1361	6332.5419	1028.3439	139.9852	6.642 × 10^−6^
	6	89.8033333	6.172	11.99	88.5	0.1355	6333.9524	1026.2399	139.0352	7.0949 × 10^−6^
15	1	86.2633333	10.614	12.01	70.3	0.1708	5844.4326	550.6343	94.0700	1.063 × 10^−5^
	2	86.33	10.614	12	71.4	0.1681	5853.4695	551.4857	92.6867	1.0789 × 10^−5^
	3	86.4533333	10.646	12.01	71.5	0.1680	5870.2063	551.4002	92.6198	1.0797 × 10^−5^
	4	89.89	6.168	12	89.3	0.1344	6346.1837	1028.8884	138.2605	7.2327 × 10^−6^
	5	89.8833333	6.3	12	86.5	0.1387	6345.2425	1007.1813	139.7246	7.1569 × 10^−6^
	6	89.9066667	6.364	12	87.3	0.1375	6348.5373	997.5703	137.1231	7.2927 × 10^−6^
20	1	86.2833333	10.288	11.99	73.2	0.1638	5847.1429	568.3459	93.0938	1.0742 × 10^−5^
	2	86.2233333	10.09	11.99	76	0.1578	5839.0138	578.6931	91.2965	1.0953 × 10^−5^
	3	86.2733333	10.67	11.98	74.1	0.1617	5845.7877	547.8714	88.5762	1.129 × 10^−5^
	4	89.9733333	6.226	11.97	85.4	0.1402	6357.9558	1021.1943	143.1346	6.9864 × 10^−6^
	5	89.9366667	6.154	11.97	88	0.1360	6352.7747	1032.3001	140.4163	7.1217 × 10^−6^
	6	89.7966667	6.388	11.98	83.4	0.1436	6333.0120	991.3920	142.4086	7.022 × 10^−6^
25	1	86.2766667	9.91	11.98	76.6	0.1564	5846.2394	589.9333	92.2637	1.0838 × 10^−5^
	2	86.27	10.474	11.98	75.8	0.1580	5845.3360	558.0806	88.2032	1.1337 × 10^−5^
	3	86.2733333	9.666	11.98	79.6	0.1505	5845.7877	604.7784	91.0207	1.0987 × 10^−5^
	4	89.8866667	6.034	11.97	95	0.1260	6345.7131	1051.6594	132.5091	7.5467 × 10^−6^
	5	89.9033333	5.832	11.97	92.5	0.1294	6348.0665	1088.4888	140.8563	7.0994 × 10^−6^
	6	89.89	5.676	11.97	91.5	0.1308	6346.1837	1118.0732	146.2660	6.8369 × 10^−6^

## Appendix B

Av-PLAT-7 technical sheet, by Avanzare [15]:
